# Restrictercise! Preferences Regarding Digital Home Training Programs during Confinements Associated with the COVID-19 Pandemic

**DOI:** 10.3390/ijerph17186515

**Published:** 2020-09-07

**Authors:** Jan Wilke, Lisa Mohr, Adam S. Tenforde, Pascal Edouard, Chiara Fossati, Marcela González-Gross, Celso Sanchez Ramirez, Fernando Laiño, Benedict Tan, Julian David Pillay, Fabio Pigozzi, David Jimenez-Pavon, Bernhard Novak, David Url, Mandy Zhang, Mireille van Poppel, Christoph Heidt, Steffen Willwacher, Lutz Vogt, Evert Verhagen, Karsten Hollander, Luiz Hespanhol, Gustavo Yuki

**Affiliations:** 1 Department of Sports Medicine, Goethe University Frankfurt, 60488 Frankfurt/Main, Germany; mohr@sport.uni-frankfurt.de (L.M.); l.vogt@sport.uni-frankfurt.de (L.V.); 2 Department of Physical Medicine and Rehabilitation, Spaulding Rehabilitation Hospital, Harvard Medical School, Charlestown, 02129 MA, USA; atenforde@mgh.harvard.edu (A.S.T.); karsten.hollander@medicalschool-hamburg.de (K.H.); 3 Inter-University Laboratory of Human Movement Science, University of Lyon, University Jean Monnet, 42000 Saint Etienne, France; pascal.edouard@univ-st-etienne.fr; 4 Department of Clinical and Exercise Physiology, Sports Medicine Unity, University Hospital of Saint-Etienne, Faculty of Medicine, CEDEX 2, 42055 Saint-Etienne, France; 5 Department of Movement, Human and Health Sciences, University of Rome “Foro Italico”, 00135 Rome, Italy; chiara.fossati@uniroma4.it (C.F.); fabio.pigozzi@uniroma4.it (F.P.); 6 ImFine Research Group, Department of Health and Human Performance, Universidad Politécnica de Madrid, 28040 Madrid, Spain; marcela.gonzalez.gross@upm.es; 7 Exercise is Medicine Spain, 28040 Madrid, Spain; david.jimenez@uca.es; 8 School of Physical Activity Sciences, University of Santiago de Chile, 8320000 Santiago de Chile, Chile; celso.sanchez@usach.cl; 9 Fundación Instituto Superior de Ciencias de la Salud, 1406 Buenos Aires, Argentina; fernandoalainio@gmail.com; 10 Changi General Hospital, Singapore 529889, Singapore; benedict.tan.c.l@singhealth.com.sg (B.T.); mandy.zhang.j@singhealth.com.sg (M.Z.); 11 Durban University of Technology, Durban 4001, South Africa; pillayjd@dut.ac.za; 12 Department of Physical Education, MOVE-IT Research Group, Faculty of Education Sciences University of Cádiz, 11519 Puerto Real, Spain; 13 Institute of Human Movement Science, Sport and Health, University of Graz, 8010 Graz, Austria; bernhard.novak@uni-graz.at (B.N.); david.url@edu.uni-graz.at (D.U.); Mireille.van-poppel@uni-graz.at (M.v.P.); 14 Department of Orthopedics, University Children’s Hospital Basel, University of Basel, 4056 Basel, Switzerland; christoph.heidt@ukbb.ch; 15 School of Human Movement and Nutrition Sciences, University of Queensland, St Lucia, QLD 4067, Australia; s.willwacher@dshs-koeln.de; 16 Institute of Biomechanics and Orthopaedics, German Sport University Cologne, 50933 Cologne, Germany; 17 Amsterdam Collaboration on Health & Safety in Sports, Department of Public and Occupational Health, Amsterdam Movement Sciences, Amsterdam UMC, University Medical Centers-Vrije Universiteit Amsterdam, 1105 Amsterdam, The Netherlands; e.verhagen@amsterdamumc.nl (E.V.); l.hespanhol@outlook.com (L.H.); 18 Faculty of Medicine, MSH Medical School Hamburg, 20457 Hamburg, Germany; 19 Masters and Doctoral Programs in Physical Therapy, Universidade Cidade de São Paulo (UNICID), Sao Paulo 03071-000, Brazil; gustavo.yuki@hotmail.com

**Keywords:** physical activity, COVID-19, coronavirus, telemedicine, e-Health

## Abstract

Confinement measures during the COVID-19 pandemic have caused substantial reductions in global physical activity (PA) levels. In view of the manifold health benefits of PA, the development of interventions counteracting this trend is paramount. Our survey with 15,261 participants (38 ± 15 years, 58.5% females) examined preferences towards digital home exercise programs in 14 countries affected by COVID-19. More than two-thirds of the sample (68.4%, *n* = 10,433) indicated being interested in home exercise, and most participants were willing to work out at least three times per week (89.3%, *n* = 9328). Binary logistic regression revealed that female sex, working part-time, younger age, and being registered in a gym were associated with willingness to exercise. Flexibility (71.1%, *n* = 7377), resistance (68.6%, *n* = 7116), and endurance training (62.4%, *n* = 6478) were the most preferred types of exercise. Our results may guide health providers in developing individually tailored PA interventions during the current and future pandemics.

## 1. Introduction

The spread of the novel coronavirus, also referred to as COVID-19, has prompted countries worldwide to restrict public life over weeks to months. Investigations into the effectiveness of related measures demonstrate that confinement strategies effectively curbed the pandemic [1,2]. However, controlling the contagion by means of lockdowns could have negative implications for health. A recent population-based survey recruiting 13,503 participants from five continents revealed a 41% decline in physical activity (PA) compared to pre-restrictions [3]. On the basis of data collected in China, it was estimated that the portion of insufficiently active individuals in China tripled during the early phase of the pandemic [4].

According to the literature, inactivity causes 9% of premature mortality, and reducing it by only 10% could avert more than 500,000 deaths per year [5]. The impact of the current PA decreases may, therefore, have detrimental consequences. With gyms, sports clubs, and other public activity spaces rendered inaccessible, the development of alternative movement opportunities is paramount. Tele-exercise represents a cost-effective and easy-to-distribute option for individuals mandated to stay at home [6]. Previous research demonstrated that meeting the individual preferences of the target population represents a key aspect to consider in the design of new PA offers. For instance, older adults’ adherence to fall prevention programs is related to the inclusion of specific components (e.g., balance exercise) rather than to the general effectiveness of the interventions [7]. In a similar way, back and neck pain patients are more compliant with home exercise when positively evaluating the program characteristics [8]. Against this background, the present study examined the preferences towards digital home exercise programs in individuals affected by the COVID-19 pandemic.

## 2. Materials and Methods

### 2.1. Ethical Standard and Study Design

The cross-sectional ASAP (Activity and health during the SARS-CoV-2 pandemic) survey [9] was performed in 14 countries (Argentina, Australia, Austria, Brazil, Chile, France, Germany, Italy, the Netherlands, Singapore, South Africa, Spain, Switzerland, and the United States of America (USA)). Approval was obtained from the study center’s ethics committee (Local Ethics Committee of the Faculty of Sports Sciences and Psychology, ref. 2020-13) and the ethics committees of the other collaborating partners. All participants provided informed consent. 

### 2.2. Participants

Individuals aged 18 and older from countries with (1) official cases of COVID-19 and (2) governmental restrictions limiting movement in public spaces were eligible. Recruitment included social media promotions (e.g., Facebook), mailing lists, and health-related multipliers (e.g., national “Exercise is Medicine” chapters).

### 2.3. Questionnaire

The herein reported part of the ASAP survey measured the participants’ preferences regarding digital home exercise programs delivered via internet. In addition to ascertaining the general willingness to participate in related programs (yes/no), the optimal duration (free entry, min/week), training frequency (workouts per week; 1–2, 3–4, 4–6 or daily), and exercise types (flexibility, resistance, endurance, balance/stability, cognition, relaxation) were assessed. Additional information obtained from other sections of the ASAP survey included age, sex, work mode (home office/office/both), and volume (part-time/full-time), as well as physical activity guideline compliance (yes/no; ≥150 min moderate, ≥75 min vigorous PA or an adequate combination of both as per the World Health Organization (WHO), assessed using the Nordic Physical Activity Questionnaire, short version [10]). 

### 2.4. Data Processing and Statistics

Data are presented as mean and standard deviation (SD), median and interquartile range (IQR), or absolute and relative frequency, as appropriate. Factors influencing (a) the willingness to exercise and (b) the preference of specific components (e.g., resistance or endurance exercise) were investigated using multiple binary logistic regression. The results were presented as adjusted odds ratios (OR) and 95% confidence intervals (CI). Calculations were made with SPSS 22 (SPSS Inc., Chicago, IL, USA). 

## 3. Results

A total of *n* = 15,261 responses (Argentina: *n* = 1021, Australia: *n* = 325, Austria: *n* = 806, Brazil: *n* = 1800, Chile: *n* = 1364, France: *n* = 2433, Germany: *n* = 2294, Italy: *n* = 903, Netherlands: *n* = 203, Singapore: *n* = 941, South Africa: *n* = 658, Spain: *n* = 632, Switzerland: *n* = 429, USA: *n* = 1193, others: *n* = 259) were obtained. Mean age was 38 (SD 15) years and 58.9% (*n* = 8935) were females. 

### 3.1. Exercise Preferences

Over two-thirds of the participants (68.4%, *n* = 10,433) indicated readiness to engage in digital home exercise. Among these, the chosen duration (median) was 40 (IQR: 30–60) minutes per session. The majority of the participants preferred working out at least three times weekly (89.3%, *n* = 9328). The most popular contents were flexibility (71.1%, *n* = 7377), resistance (68.6%, *n* = 7116), and endurance exercise (62.4%, *n* = 6478), while relaxation (42.6%, *n* = 4416) and cognitive training (24.2%, *n* = 2514) were selected less frequently. 

### 3.2. Variable Associations

Logistic regression revealed four factors associated with interest in digital home exercise: female sex, working part-time, younger age, and having exercised in a gym pre-restrictions (Table 1). 

With regard to exercise types (Table 2), older participants (≥40 years) were more likely to select flexibility and less likely to choose resistance, endurance, and cognitive training. Marked differences also occurred between men and women. Female sex was associated with a more frequent choice of flexibility and relaxation exercises and a less frequent selection of resistance, cognitive, and endurance exercise. Participants with high physical activity levels (meeting WHO PA recommendations) more often preferred resistance, endurance, and balance/stability training, but not other forms of exercise. Type of employment (full-time/part-time) was weakly/not associated with exercise preference. In most cases, individuals working remotely (home office) had comparable odds to participants working outside the home (in the office). However, individuals who combined working at home and in the office had a higher preference of most exercise types than persons working outside the home only. Not having a formal employment was associated with a less frequent choice of resistance and endurance training but more frequent choice of balance/stability, cognitive, flexibility, and relaxation exercise.

## 4. Discussion

A wealth of evidence supports the manifold health benefits of sufficient and regular engagement in physical activity [11]. Not only because of these general effects, but also because exercise can have a positive impact on immune function and reduce upper respiratory tract infections [12], researchers have underlined the need to maintain or improve PA habits during mandated lockdowns [13,14]. To the best of our knowledge, the present study is the first to describe the exercise preferences of individuals affected by the COVID-19 pandemic. More than two in three participants indicated willingness to engage in digital home exercise programs. This particularly applied to women whose odds of being interested were 1.7 times higher than those of men. On the whole, our data suggest that tele-health interventions could be well received, thereby helping to stem any reduced PA during confinements [3]. Reports from China indicate that public life restrictions caused considerable increases in anxiety and depression [15]. As exercise is effective in addressing both, supporting the maintenance of regular PA may be crucial not only for physical health, but also for mental well-being. 

Although abundant evidence underlines the relevance of matching program design and participant preferences in special populations such as patients, the elderly, or postmenopausal women [8,9], there is a paucity of studies investigating the preferences of asymptomatic individuals. This report provides significant information toward supporting tailored programs on the basis of specific needs of different target groups during the COVID-19 pandemic. For example, new programs should have a minimum frequency of three sessions per week. This is in line with statements of the American College of Sports Medicine [16] recommending resistance, flexibility, and neuromotor training 2–3 times weekly and cardiorespiratory training 3–5 times weekly.

Flexibility training was the most preferred exercise type, followed by resistance and endurance training. Benefits of flexibility exercise include the promotion of well-being and relaxation [17]. Therefore, its choice could be an attempt to minimize the psychological impact caused by public life restrictions. Furthermore, flexibility exercise does not require extensive space or equipment, making it easy to perform at home and potentially more attractive than other forms of training. However, exercise preference varied considerably as a function of sex and age. Whereas women presented a stronger orientation to flexibility and relaxation, men were more interested in endurance, resistance, and cognition. The latter three were also more popular among younger vs. older participants who rather seemed to require flexibility. The observed patterns might be explained by the belief that resistance and endurance exercise could be more “vigorous” than flexibility and/or relaxation exercises. The perceived “safety” (e.g., in terms of injuries during training) might, hence, influence exercise preference. This particularly applies to women/older participants who display a higher health perception and are more conservative with regard to healthy behaviors than men and younger individuals [18,19]. While future studies should test this hypothesis, we suggest calibrating the exercise modality and intensity to the risk appetite of each group, in order to encourage compliance. 

Finally, another remarkable finding was that active participants had more than twice the odds of preferring resistance exercises and about 1.5 the odds of preferring endurance training. Seeking to improve performance, they may prefer vigorous exercises, while less active individuals may select less vigorous exercises aiming to acquire health benefits with the lowest possible risk of adverse events.

Some limitations have to be discussed. Firstly, this was an internet survey, and promotion was mainly based on social media promotion. Persons with limited or no internet access and individuals with small affinity for digital content may, therefore, have had a lower chance to participate. Another issue relates to the items included. Although the questions were mostly self-explanatory, a few contents could be interpreted differently. For instance, some participants may have assigned yoga and light stretching/mobility training to “relaxation exercise”. while others may have understood the term as only describing specific techniques such as progressive muscle relaxation. Finally, while we examined important program characteristics such as the exercise type, training frequency, and session duration, we did not include preferred intensity, which would have been interesting as it may moderate the protective effect of exercise against viral infections.

## 5. Conclusions

In summary, a large portion of individuals affected by confinements related to the COVID-19 pandemic are interested in digital home exercise. Interventions meeting their needs should consider factors such as the frequency (minimum: three times a week), duration (40 min), and type (flexibility, resistance, endurance) of program. Additionally, carefully balancing the different needs of individuals, such as old versus young, male versus female, and active versus inactive, is recommended.

## Figures and Tables

**Table 1:** Variables associated with willingness to participate in online home exercise programs.

Variable	Descriptive Statistics	OR (95% CI)
**Age, % (*n*)**		
<40 years	62.2 (9488)	Reference
≥40 years	37.8 (5773)	0.707 (0.619 to 0.808) *
**Sex, % (*n*)**		
Male	41.1 (6237)	Reference
Female	58.9 (8935)	1.747 (1.577 to 1.936) *
**Physical Activity Level, % (*n*)**		
Not meeting guidelines	18.5 (2423)	Reference
Meeting guidelines	81.5 (10,681)	0.924 (0.831 to 1.027)
**Exercising in a Gym, % (*n*)**		
No	61.1 (9323)	Reference
Yes	38.9 (5938)	1.324 (1.188 to 1.476) *
**Exercising in a Sports Club, % (*n*)**		
No	70.9 (10,821)	Reference
Yes	29.1 (4440)	1.093 (0.972 to 1.229)
**Exercising Self-Organized, Indoor, % (*n*)**		
No	74.4 (11,360)	Reference
Yes	25.6 (3901)	0.954 (0.847 to 1.074)
**Exercising Self-Organized, Outdoor, % (*n*)**		
No	40.4 (6164)	Reference
Yes	59.6 (9097)	0.973 (0.873 to 1.084)
**Work Mode, % (*n*)**		
Office	16.3 (2439)	Reference
Home office	44.2 (6600)	1.024 (0.917 to 1.143)
Home office and office	11.5 (1714)	1.073 (0.927 to 1.241)
No formal employment	28.0 (4185)	0.942 (0.836 to 1.061)
**Working Volume, % (*n*)**		
Full-time	66.8 (5600)	Reference
Part-time	33.2 (2781)	1.249 (1.118 to 1.394) *

The adjusted ORs were estimated by the multiple binary logistic regression model. SD: standard deviation. OR: adjusted odds ratio for all independent variables included in the model; CI: confidence interval. * Statistically significant.

**Table 2:** Associations between preferred workout contents and sample characteristics.

	Dependent Variables					
Independent Variables	Resistance OR (95% CI)	Endurance OR (95% CI)	Balance/Stability OR (95% CI)	Cognition OR (95% CI)	Flexibility OR (95% CI)	Relaxation OR (95% CI)
**Age**						
<40 years	Reference	Reference	Reference	Reference	Reference	Reference
≥40 years	0.470 (0.414 to 0.533) *	0.619 (0.550 to 0.696) *	1.075 (0.960 to 1.203)	0.822 (0.718 to 0.940) *	1.375 (1.213 to 1.559) *	0.960 (0.855 to 1.077)
**Sex**						
Male	Reference	Reference	Reference	Reference	Reference	Reference
Female	0.650 (0.568 to 0.742) *	0.789 (0.697 to 0.893) *	1.070 (0.951 to 1.205)	0.822 (0.715 to 0.945) *	1.445 (1.270 to 1.645) *	1.610 (1.425 to 1.819) *
**Physical Activity Level**						
Not meeting guidelines	Reference	Reference	Reference	Reference	Reference	Reference
Meeting guidelines	2.104 (1.810 to 2.447) *	1.486 (1.282 to 1.722) *	1.347 (1.165 to 1.558) *	1.004 (0.843 to 1.195)	1.101 (0.937 to 1.294)	0.655 (0.566 to 0.759) *
**Work Mode**						
Office	Reference	Reference	Reference	Reference	Reference	Reference
Home office	0.934 (0.815 to 1.070)	0.871 (0.766 to 0.990) *	1.037 (0.918 to 1.171)	1.152 (0.994 to 1.335)	1.113 (0.973 to 1.273)	1.225 (1.081 to 1.388) *
Home office and office	1.228 (1.023 to 1.475) *	1.026 (0.866 to 1.217)	1.209 (1.028 to 1.422) *	1.221 (1.008 to 1.479) *	1.095 (0.916 to 1.311)	1.243 (1.055 to 1.466) *
No formal employment	0.752 (0.649 to 0.871) *	0.771 (0.671 to 0.886) *	1.145 (1.003 to 1.307) *	1.366 (1.168 to 1.597) *	1.178 (1.018 to 1.363) *	1.260 (1.110 to 1.443) *
**Work Volume**						
Full-time	Reference	Reference	Reference	Reference	Reference	Reference
Part-time	0.992 (0.870 to 1.129)	1.124 (0.994 to 1.271)	1.099 (0.976 to 1.236)	1.261 (1.098 to 1.449) *	0.923 (0.810 to 1.052)	1.127 (1.000 to 1.271) *

The adjusted ORs were estimated by the multiple binary logistic regression model. OR: adjusted odds ratio for all independent variables included in the models; CI: confidence interval. * Statistically significant.
